# Abnormal Cerebellar Development Is Involved in Dystonia-Like Behaviors and Motor Dysfunction of Autistic BTBR Mice

**DOI:** 10.3389/fcell.2020.00231

**Published:** 2020-04-07

**Authors:** Rui Xiao, Hongyu Zhong, Xin Li, Yuanyuan Ma, Ruiyu Zhang, Lian Wang, Zhenle Zang, Xiaotang Fan

**Affiliations:** ^1^Department of Military Cognitive Psychology, School of Psychology, Army Medical University, Chongqing, China; ^2^Department of Basic Nursing, School of Nursing, Army Medical University, Chongqing, China

**Keywords:** autism spectrum disorders, dystonia, movement disorder, cerebella, neurodevelopment

## Abstract

Motor control and learning impairments are common complications in individuals with autism spectrum disorder (ASD). Abnormal cerebellar development during critical phases may disrupt these motor functions and lead to autistic motor dysfunction. However, the underlying mechanisms behind these impairments are not clear. Here, we utilized BTBR *T^+^ Itpr^tf^*/J (BTBR) mice, an animal model of autism, to investigate the involvement of abnormal cerebellar development in motor performance. We found BTBR mice exhibited severe dystonia-like behavior and motor coordination or motor learning impairments. The onset of these abnormal movements coincided with the increased proliferation of granule neurons and enhanced foliation, and Purkinje cells displayed morphological hypotrophy with increased dendritic spine formation but suppressed maturation. The migration of granule neurons seemed unaffected. Transcriptional analyses confirmed the differential expression of genes involved in abnormal neurogenesis and revealed *TRPC* as a critical regulator in proliferation and synaptic formation. Taken together, these findings indicate that abnormal cerebellar development is closely related to dystonia-like behavior and motor dysfunction of BTBR mice and that *TRPC* may be a novel risk gene for ASD that may participate in the pathological process of autistic movement disorders.

## Introduction

Autism spectrum disorder (ASD) is a pervasive neurodevelopmental disorder characterized by persistent defects in social communication and interaction and restricted, repetitive, inflexible behaviors and interests (Lord et al., 2018). It is commonly diagnosed in early childhood, and the prevalence of ASD has increased dramatically throughout the last decades (Constantino and Charman, 2016). The interaction of genetic and environmental factors was recently hypothesized to contribute to the pathogenesis of ASD (Ecker et al., 2015; Richards et al., 2015), but the etiology of the disorder remains far from clear.

In addition to the core symptoms, autistic subjects frequently present with complex motor impairments, such as ataxia, dystonia, and akinesia (Cook, 2016). Notably, a systematic research on the prevalence of movement disorders in ASD associated with specific genetic syndromes revealed 43.6–100% for ataxia and 25.0–48.3% for tremor, with additional reports for dystonia and rigidity (Bell et al., 2019). Several clinicians have proposed that these atypical movement impairments are a predictor for ASD, because these impairments often appear prior to the classical behaviors of ASD (Robledo et al., 2012; Uljarevic et al., 2017). Motor disturbances may underlie some of the behavioral core features in autism. The contribution of movements to social cognition and cascade effects on social communication in individuals with autism have been reported (Cook, 2016; Baranek et al., 2018). Notably, therapeutic medicines for motor dysfunction, such as methylphenidate and atomoxetine, have improved social interaction deficits and recognition memory impairment in ASD subjects (Hara et al., 2016). It is quite essential and meaningful to illuminate the relationship between autism and motor dysfunctions to provide comprehensive and precise treatment. Several hypotheses have been raised, but more evidence is needed, and the shared underlying mechanisms with autism should be further examined.

Several brain regions have been implicated in the pathogenesis of ASD, but cerebellar abnormalities are the most reproducibly studied in this disorder. Neuropathological studies showed lower Purkinje cell (PC) numbers, missed or ectopic neurons of deeper cerebellar nuclei (DCN), cortical thickness alterations, foliation dysplasia and migration impairments in the cerebellar cortex of individuals with ASD (Amaral et al., 2008; Wegiel et al., 2010, 2014; Blatt, 2012). Indeed, cerebellar lesions are associated with increased rates of autistic behavior, and recent evidence has suggested more to be involved, like suppressed social function, restrictive or repetitive behaviors, and motor impairment symptoms such as ataxia, dystonia and tremor (Jaber, 2017). It was recently proposed that early perinatal alterations of the cerebellum are involved in ASD pathogenesis, which is supported by the finding that autism genes are frequently involved in the aberrant cerebellar development (Menashe et al., 2013).

The cerebellum is characterized by a typical laminated structure consisting of a molecular layer (ML), a Purkinje cell layer (PCL) and an inner granule layer (IGL) (Xu et al., 2013). During cerebellar development, billions of granule neurons are produced in the external granule layer (EGL) and then descend and migrate to destinations in the IGL, leaving “T-shaped” parallel fibers that are arranged in parallel along the cerebellar folia axis and synapse on the dendritic arbors of PCs (Altman and Bayer, 1978; Hampson and Blatt, 2015). The PC axons further travel to the DCN and project more broadly. As the sole efferent neurons in the cerebellum, Purkinje neurons regulate all of the information transfer and are responsible for cerebellar function (Martinez et al., 2013). Notably, the loss of cerebellar PCs is one of the most consistent findings in postmortem studies of autistic cerebella (Fatemi et al., 2012). Therefore, a further raised question is what extended morphological aberrations of the cerebellum concomitantly occur in specific motor impairment.

The BTBR T^+^ Itpr3^tf^/J (BTBR) inbred strain shows a robust behavioral phenotype that mimic core symptoms of ASD patients and exhibits striking anatomical features in the cerebellum. We investigated the contribution of abnormal cerebellar development to movement disorders in BTBR mice, with the control of C57BL/6J strain (commonly used as wild-type, WT). The present study revealed distinct dystonia-like behaviors and motor learning impairments in BTBR mice that began in early postnatal days. Concomitant with the progression of behavioral impairment, a hyperplastic cerebellum with enhanced foliation was identified due to the abnormally increased proliferation of granule cell precursors (GCPs). Moreover, in the cerebellum of BTBR mice, the morphology of Purkinje neurons was altered and exhibited hypotrophy and disturbed spine formation. Evidence from RNA sequencing indicated that the nervous system development was negatively regulated, and the transient receptor potential canonical channel (*TRPC*) family including *TRPC6*, *TRPC3* and *TRPC4* played a key role in the signal regulation of the abnormal neurogenesis. Together, these findings suggest that abnormal cerebellar development, which may be regulated by *TRPC*, was involved in the pathological progression from movement disorders to autism.

## Materials and Methods

### Animals

BTBR mice were originally obtained from Jackson Laboratory (BTBR *T*^+^ *Itpr*3*^tf^*/J; stock number 002282) and maintained in our mouse colony at the Army Medical University. C57BL/6J mice (WT) were used as controls and provided by the Army Medical University. Only male mice were used in the experiments. After weaning at 3 weeks, mice were group-housed with 4–6 mice per cage under a controlled environment (22 ± 2°C, 45 ± 10% humidity, 12 h light/dark cycle) with free access to water and food. The Army Medical University approved all experiments, which were performed according to the accepted standards of animal care. Efforts were made to reduce animal suffering.

### Behavioral Assays

Mice were examined periodically using the tail suspension test and grid hang test from early postnatal (P) days to 5 months. Other behavioral assessments, such as horizontal ladder rung walking, rotarod and open field tests, were initiated at 8 weeks.

### Tail Suspension Test

Mice were suspended by their tails for 60 s. The activity of the mice was recorded by a camera to observe the presence of dystonia-like behaviors, as references described (Liu et al., 2015; Pappas et al., 2015), including hyperflexion, trunk twisting, hyperextension, and forelimb or hindlimbs clasping. These phenotypes were recorded from P3 to P150.

### Grid Hang Test

Motor coordination and strength were detected by putting the mice on a 30 × 50 cm wire grid with 0.5 cm^2^ openings. The grid was inverted after the mouse grabbed the grid with its fore- and hindlimbs, and the latency to fall off was recorded. Movement on the grid and the paw placement of the mice were observed during the test.

### Horizontal Ladder Rung Walking Test

Skilled fore- and hindlimb coordination and fine motor function were assessed by the horizontal ladder rung walking test (Metz and Whishaw, 2002). To subtly test coordination, the difficulty of the task was divided into two patterns: pattern A used a regular rung arrangement with 2 cm intervals, and pattern B used an irregular rung pattern with randomly spaced rungs at intervals from 1 to 3 cm. Mice were put on one side of the ladder (30 cm above the ground) and allowed to freely cross it. The whole crossing process was filmed with a high-definition video camcorder (Logitech C930e), and behaviors were analyzed. The total limb falls and time to cross the ladder were recorded to assess motor function. Five trials were performed on each mouse, and the average value was calculated.

### Accelerating Rotarod Test

Motor coordination and motor learning were detected using the accelerating rotarod test. Mice were habituated to stay on the stationary drum for 3 min 1 day in advance and habituated was repeated every day for 1 min just before the session. Once stabilized, mice were put on an accelerating rod (3 cm diameter, 14 cm above the pedestal), and the speed was set to 5 rpm with a uniform acceleration to 40 rpm in 5 min. The latency of mouse falling from the rotating rod was calculated. Five trials were performed on each mouse per day for 5 consecutive days to assess motor learning in each group.

### Open Field Locomotion Test

Mice were put in an open field to assess locomotor activity. The apparatus was a square plexiglass cage (40 × 40 × 30 cm) illuminated at ∼200 lux. As previously described (Cai et al., 2017), square grid lines were predefined by a computer that divided the open field chamber into a central zone and periphery. Mice were placed in the center of the apparatus, and locomotion was traced for 30 min using Ethovision 11.0 software (Noldus). The total distance in all zones and the center zone were calculated to assess the activity of mice. The apparatus was cleaned with 70% ethanol between trials.

### Histology and Immunohistochemistry

Adult mice were completely anesthetized with sodium pentobarbital and transcardially perfused with ice-cold 0.01 M phosphate-buffered saline (PBS) followed by 4% paraformaldehyde (PFA) in PBS. Neonatal mice received BrdU injection (50 mg/kg i.p.) and then were decapitated 2 h later, and the needed tissues were dissected. Dissected cerebella were postfixed in 4% PFA for 48 h and dehydrated in a 50–100% gradient alcohol series. Tissues were soaked twice in trichloromethane for 20 min and covered twice with melted paraffin for 30 min. Cerebella were paraffin-embedded, and then sagittal sections (5 μm) were collected. All sections used in each mouse were taken from the same medial lateral position on the cerebellum to allow comparisons and five sections per mouse were used for each staining. Paraffin sections were performed dewaxing and antigen retrieval before used. For hematoxylin-eosin (HE) staining, sections were incubated in hematoxylin (ZLI-9610, ZSJQ-Bio, China) for 10 min, washed in running water, differentiated by 75% hydrochloric acid in alcohol for 1 min, washed in running water when the nucleus was black to blue, incubated in eosin (ZLI-9612, ZSJQ-Bio, China) for 10 s, dehydrated in 95% alcohol for 1 min, and mounted in DPX (06522, Sigma, United States). For immunohistochemical staining, sections were processed by washing in 0.3% Triton-X/PBS, blocking in 3% bovine serum albumin (BSA) (37°C, 2 h), and incubation with the following primary antibodies (room temperature (RT), overnight): (1) mouse anti-5-bromo-2′-deoxyuridine (BrdU) (1:500, BD PharmingenTM, United States); (2) rabbit anti-Ki67 (1:1000, Thermo, United States); (3) rabbit anti-neuronal nuclei (NeuN) (1:200, Abcam, United States); rabbit anti-s100β (1:500, CST, United States); rabbit anti-glial fibrillary acidic protein (GFAP) (1:500, Dako, Japan); and mouse anti-calbindin (CB) (1:1000, Swant, Switzerland). After washing in 0.01 M PBS for 30 min, sections were incubated with the corresponding secondary antibodies (RT, 3 h in darkness): Alexa Fluor 488-conjugated donkey anti-mouse IgG (1:500, Jackson ImmunoResearch, United States); and cy3-conjugated donkey anti-rabbit IgG (1:500, Jackson ImmunoResearch, United States). 4′,6-Diamidino-2-phenylindole (DAPI) (1:10000, Sigma-Aldrich, United States) was used to counterstain the nuclei in all sections. After air drying, sections were mounted with Vectashield (Vector Lab, United States). Sections for Nissl staining were incubated in a cresyl violet solution containing acetic acid (C0117, ZSJQ-Bio, China) (37°C, 30 min), dehydrated in 95% alcohol for 1 min, and mounted in DPX (06522, Sigma, United States). The stained sections were observed under 5X or 20X objective lens using a Zeiss Axiovert microscope (Oberkochen, Germany) with a Zeiss Axiovision 4.0 system.

### Golgi Staining

Mice were decapitated immediately at P14, and brains were dissected. Cerebella were rinsed and processed for Golgi staining with the FD Rapid Golgi Stain Kit (PK401A, FD Neurotechnologies, United States) according to the manufacturer’s protocol. Sagittal sections (80 μm) were generated and mounted in DPX after drying. Slides were observed under 20X or 100X oil objective lens using a Zeiss Axiovert microscope. Sholl analysis was executed using the matched Zeiss Axiovision 4.0 system.

### Western Blot

P14 BTBR and WT mice were decapitated, and their cerebella were dissected in ice-cold PBS. The total protein was extracted immediately, and protein concentrations were measured using a Bicinchoninic Acid Kit (Beyotime, China) as previously described (Xiao et al., 2017). The total protein (20 μg) of each sample was separated by 10% SDS-polyacrylamide electrophoresis (80 V, 100 min) and then transferred to a polyvinylidene fluoride (PVDF) membrane (220 mA, 60 min). The membranes were washed in 1% Tween-20/Tris-buffered saline (TBS) (TBS-T), blocked in 5% BSA/TBS-T (RT, 2 h), and incubated with a primary antibodies (4°C, 12 h) (1) mouse anti-CB (1:2000, Swant, Switzerland) and (2) mouse anti-GAPDH (1:2000, Cell Cwbio, China), followed by peroxidase-conjugated goat anti-mouse secondary antibody IgG (1:1000, Santa Cruz Biotechnology, United States). Bands were visualized using the chemiluminescence detection kit (Pierce, United States) under a Gel-Pro analyzer (Bio-Rad Laboratories, United States). Band intensity was quantified in Image Lab (Bio-Rad Laboratories, United States), and calbindin protein was normalized to GAPDH.

### Quantification

Comparable middle sagittal sections were selected for assessments. Cerebella sagittal area (mm^2^) and perimeter (mm) were defined as shown in Figure 2E and calculated with a Zeiss Axiovision 4.0 system. Lobes separated clearly by fissures were calculated as the lobe number. BrdU- and Ki67-positive granule cell precursors were counted only in the EGL and the density was calculated as total BrdU or Ki67 cells in the EGL/EGL area, and proportion of BrdU-positive cell in the EGL was calculated as BrdU-positive cells/DAPI-stained nuclei in a high-power field of the EGL (218 μm length) in lobe IV/V. Bergmann soma in the PCL and fibers in the ML were counted under a 20X objective lens along the lobe axis (450 μm length), and the densities were calculated. Purkinje neuron density and soma size were counted in each lobe along a 500 μm-length axis in the middle of each lobe. Among these cells, 10 Purkinje neurons were randomly selected, and their soma areas were measured by a Zeiss Axiovision 4.0 system.

For Golgi staining quantification, the outer terminals of the Purkinje dendritic branches were orderly linked, and the formative closed region was defined as the PC dendritic area. Primary dendrite length was measured as the primary dendrite of each Purkinje neuron from the soma up to the end at the surface of the ML. Sholl analysis was performed, as described previously (Piochon et al., 2014). Purkinje neuron branches were incised by concentric circles with 5.5 μm radius steps from the soma, and intersections in each circle were counted. Dendrite spine density and classification were assessed referring to Risher (Risher et al., 2014). At least a 10 μm-length branch was calculated for the spine. Ten cells per mouse and 10 branches per cell were detected.

### RNA-seq Analyses

The whole cerebella of BTBR and WT mice were collected at P14 for the mRNA sequencing assay. This experiment was performed by Novogene (Beijing, China). Libraries were generated using the NEBNext^®^ UltraTM RNA Library Prep Kit for Illumina^®^ (NEB, United States) according to standard Illumina protocols. After clustering was performed in the cBot Cluster Generation System, the libraries were sequenced on an Illumina Hiseq platform with a 125 bp/150 bp paired-end reads strategy. The original image data were subjected to quality control, and reads containing adapter, poly-N and low-quality reads were removed from the raw data. Clean data of high quality were used for the downstream analyses. The reads were aligned to the reference genome (10 mm) using the split read aligner TopHat v2.0.12 and Bowtie v2.2.3, and HTSeq v0.6.1 was used to estimate the abundances of mapped genes. Expected number of Fragments Per Kilobase of transcript sequence per Million base pairs sequenced (FPKM) of each gene was calculated based on the length of the gene and reads count and used as the evaluation index of gene expression levels. For differential expression gene (DEG) analysis, the DESeq R package (1.18.0) was used to perform routine statistics with a model based on negative binomial distribution. *P*-values < 0.05 were deemed significant. Next, Gene Ontology (GO) enrichment pathway analysis of DEGs was conducted using the GOseq R package, which adjusts the gene length bias based on Wallenius hyper-distribution. GO pathways with *P*-values < 0.05 defined a significant enrichment of the DEGs. Protein-protein interaction (PPI) networks of the DEGs screened out by GO enrichment were performed in the STRING database^1^. Further analysis was performed in Cytoscape_v3.7.2 software.

### Real-Time Quantitative PCR

Cerebella were collected at P14, and total RNA was extracted using Trizol (Invitrogen, United States) and reverse transcribed to cDNA, according to the manufacturer’s protocol. Real-time PCR for target genes was performed with a SYBR Green kit (Takara Company, Japan) and the CFX Connect^TM^ Real-time system (Bio-Rad, United States). Primers are listed in the Supplementary Table. Expression of mRNA was detected via the ΔΔ cycle threshold-based algorithm relative to an internal control gene (GAPDH). Each sample was run in triplicate, and 6 mice from each group were used.

### Statistical Analysis

All data collection and analyses were performed randomly by experimenters blinded to the genotypes. The sample sizes are similar to those of previous publications (Huang et al., 2016, 2019; Xie et al., 2018) and listed in the figure legends. Data are represented as the means ± standard deviation (SD), and statistical analysis included the Chi squared test, non-parametric Mann–Whitney *U* test (for data that failed normality test), unpaired *t*-test, two-way ANOVA, and two-way repeated-measures ANOVA with *post hoc* Bonferroni multiple comparisons test were performed using SPSS 19.0 software (SPSS Inc., United States). Detailed statistical approaches and results are listed in the figure legends and Supplementary Data Sheet 1. A *P*-value < 0.05 was defined as statistically significant. In the graphed data ^∗^, ^∗∗^, and ^∗∗∗^ denote *P*-values less than 0.05, 0.01, and 0.001, respectively.

## Results

### BTBR Mice Exhibited Infancy-Onset Dystonia-Like Behaviors and Motor Impairments

**FIGURE 1:**
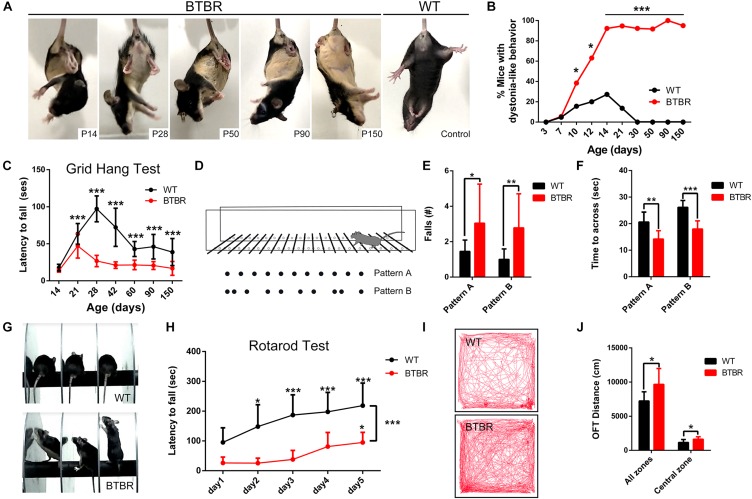
BTBR mice exhibited infancy-onset dystonia-like behavior and motor impairments. **(A)** Representative image of BTBR mice and WT control at each growing point in tail-suspension test. **(B)** Quantification of the morbidity of dystonia at each growing point showing BTBR mice developed typical dystonic behavior and aggravated with growth (Chi square test; *n* = 32, 26 mice). **(C)** Latency to fall from the wire grid at each growing point of mice, and BTBR mice developed a weaken ability to hang since adolescence (Two-way ANOVA; *n* = 15-29 mice). **(D)** The horizontal ladder rung walking apparatus with regular arrangement (pattern A) and irregular arrangement (pattern B). **(E)** Average total number of limbs fall of adult mice (8 weeks) in the horizontal ladder rung walking task (non-parametric Mann–Whitney *U* test; *n* = 10, 12 mice). **(F)** Quantification of the time to across the horizontal ladder (non-parametric Mann–Whitney *U* test, Student’s *t*-test; *n* = 10, 12 mice). **(G)** Representative images in the rotarod test showing inattention of BTBR mice. **(H)** Latency to fall from the accelerated rod of adult mice (8 weeks) showing motor and motor learning defect in BTBR mice (Two-way repeated measure test; *n* = 12, 8 mice). **(I)** Representative trace diagrams in open field test showing hyperactivity in BTBR mice. **(J)** Quantification of the distance in total and central area of adult mice (8 weeks) in the open field. (Student’s *t*-test; *n* = 9, 9 mice). All data are displayed as mean ± SD. ^∗^*P* < 0.05, ^∗∗^*P* < 0.01, ^∗∗∗^*P* < 0.001.

Movement disorders are widely reported in combination with autism in individuals. We found BTBR mice exhibited severe dystonia-like movements during tail suspension, when mice try to keep an upright body posture. The most prominent dystonic symptom was hyperflexion of one or both hyperkinetic hindlimbs during tail suspension. Hindlimb clasping or fore- and hindlimb clasping, hyperextension, and severe trunk twisting were also observed in conjunction with myodystonia (Supplementary Figure S1). BTBR mice exhibited dystonia-like behaviors as early as the tenth day (P10) (*X*^2^ = 3.902, *P* < 0.05), and nearly 100% of BTBR mice developed such behaviors beginning at P14 (*X*^2^ = 21.505, *P* < 0.001). This behavior persisted to adulthood and remained stable (Figures 1A,B). Physiological hypermyotonia was observed in WT mice between P7 and P30, but it disappeared after P30. In addition, BTBR mice also developed a defect in the ability to hang from a wire grid [Figure 1C, *F*(1,258) = 343.614, *P* < 0.001] that in some cases because of abnormal hindlimb clasping or twisting. Fine motor skill was assessed using regularly and irregularly spaced horizontal ladders at 8 weeks of age. Mice ran across the horizontal ladder in two patterns, as shown in Figure 1D. BTBR mice exhibited increased limb falls in both the regular (*U* = 26.000, P < 0.05) and irregular (*U* = 8.500, *P* < 0.01) patterns, which suggests a deficit in fine motor skill (Figure 1E). Interestingly, the time spent crossing the ladder was decreased in BTBR mice compared to WT controls (Pattern A: *U* = 9.000, *P* < 0.01; Pattern B: *T*_20_ = 6.686, *P* < 0.001) (Figure 1F), mainly because of the increased activity. BTBR mice were also significantly hyperactive in the open field, as indicated by the increased distance in all zones (*T*_16_ = 2.719, *P* < 0.05) and the central zone (*T*_16_ = 2.504, *P* < 0.05) (Figures 1I,J). Motor skill learning was assessed by means of the consecutive rotarod learning test (Figure 1H). Both BTBR and WT mice learned the task, and the time on the rod gradually increased [WT: *F*(4,44) = 21.867, *P* < 0.001; BTBR: *F*(4,28) = 15.306, *P* < 0.001]. However, learning was significantly slower in the BTBR mice [*F*(1,72) = 28.232, *P* < 0.001] (Figure 1H). Notably, the BTBR mice showed abnormal behaviors, similar as inattention, when they were put on the rod, as shown in Figure 1G. Instead of concentrating on the motor learning, the BTBR mice explored and ignored the unstable rotating rod under their feet. It may be an important factor for the impaired learning process. In summary, BTBR mice exhibited infancy-onset dystonia-like behaviors accompanied by severe deficits in motor coordination and motor learning in addition to autistic behavior.

### BTBR Mice Displayed Hyperplastic Cerebella With Increased Foliation

**FIGURE 2:**
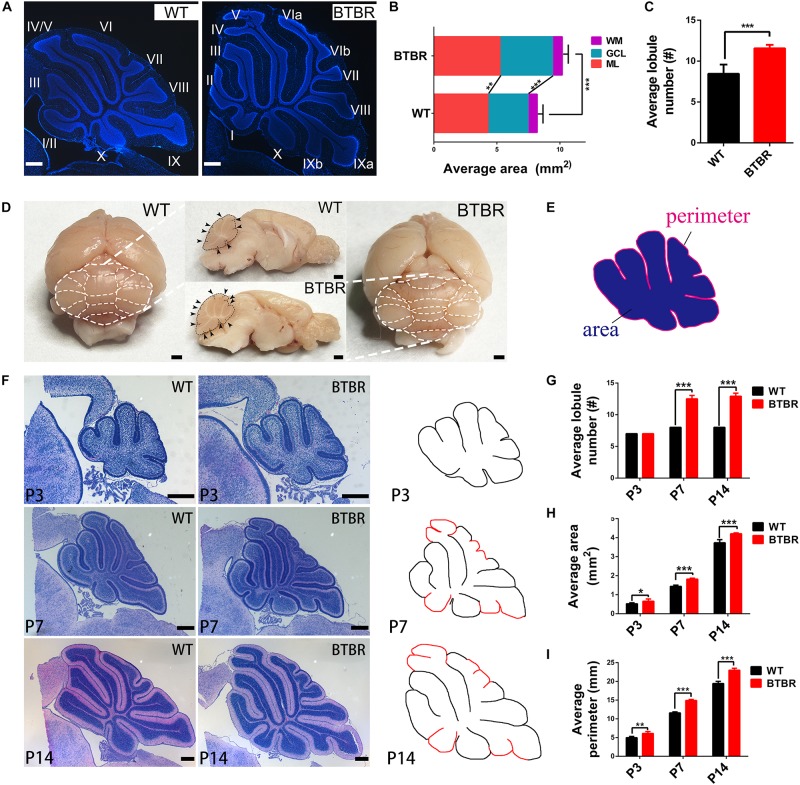
Cerebellar cortex in BTBR mice was expanded with increased foliation since post-natal. **(A)** DAPI stained cerebella of WT and BTBR mice at adult stage (P90). **(B)** Quantification of the sagittal area of cerebella and each component at adulthood (Student’s *t*-test; *n* = 6, 7 mice). **(C)** Quantification of the average lobe number showing increased foliation of adult BTBR mice (Student’s *t*-test; *n* = 6, 7 mice). **(D)** Whole mount images and sagittal section of brain in WT and BTBR mice at adult stage (P90). White and black dotted line delimit the lobule outline. Black arrowheads indicate the lobule fissures. **(E)** Schema graph illustrating the determination of perimeter and area. **(F)** Hematoxylin-eosin (HE) staining of middle sagittal cerebellar section at postnatal day 3, 7, and 14 (additional lobes highlighted in red in the counterdraw). **(G)** Quantification of the average lobule number in WT and BTBR mice at indicated stage (Student’s *t*-test; P3 *n* = 6,5; P7 *n* = 6,6; P14 *n* = 5,6). **(H)** Quantification of the average sagittal cerebellar area in WT and BTBR mice at indicated stage (Student’s *t*-test; P3 *n* = 6, 5; P7 *n* = 6, 6; P14 *n* = 5, 6). **(I)** Quantification of the average sagittal cerebellar section perimeter in WT and BTBR mice at indicated stage (Student’s *t*-test; P3 *n* = 6,5; P7 *n* = 6,6; P14 *n* = 5,6). All data are displayed as mean ± SD. *P < 0.05, **P < 0.01, ***P < 0.001. Scale bar: **(A,F)** 200 μm; **(D)** 1 mm.

In the adult BTBR mice, the overall structure of the cerebellum was abnormal, with an obviously larger area (*T*_11_ = 7.727, *P* < 0.001) and more lobules (*T*_11_ = 6.826, *P* < 0.001) (Figures 2A,D). By dividing the area into three lamellas, the ML, GCL and white matter (WM), we found that the enlarged portion was mainly in the ML (*T*_11_ = 4.383, *P* < 0.01) and GCL (*T*_11_ = 8.880, *P* < 0.001) (Figure 2B). The thickness of the ML was not altered in BTBR mice (data not shown), and its increased area might have resulted from an elongated perimeter. Therefore, the enlargement of the cerebella may be due to the extension of the GCL. Another noticeable change was observed that more foliation appeared in BTBR cerebella than the WT controls (Figure 2C). To determine when BTBR mice firstly exhibited enhanced foliation, paraffin sections of the cerebella with HE staining were detected sequentially during the first two postnatal weeks (Figure 2F). The initial stages of cerebellar patterning, including cardinal fissure formation, were normal in BTBR mice until P3, but the average sagittal cerebellar area increased significantly (*T*_9_ = 2.447, *P* < 0.05) compared to WT controls. The average sagittal cerebellar section perimeter was elongated concomitantly (*T*_9_ = 4.378, *P* < 0.01), which indicates that the cerebellar surface area was increased. Thus, cortical expansion and increased cross-sectional area preceded supernumerary folia in BTBR mice.

BTBR mice firstly exhibited increased foliation at P7, with multiple lobules that were not present in controls (*T*_10_ = 20.125, *P* < 0.001) (Figures 2F,G). Additionally, the midsagittal area (*T*_10_ = 11.234, *P* < 0.001) and perimeter (*T*_10_ = 20.698, *P* < 0.001) were increased more noticeably. At P14, when foliation patterns are established, BTBR midsagittal sections were larger (*T*_9_ = 6.739, *P* < 0.001) (Figure 2H), had a longer perimeter (*T*_9_ = 10.639, *P* < 0.001) (Figure 2I), and were considerably more foliated than controls (*T*_9_ = 22.160, *P* < 0.001) (Figures 2F,G).

### BTBR Cerebella Displayed Increased GCP Proliferation in the EGL Without Alteration in the Migration of Granule Neurons

**FIGURE 3:**
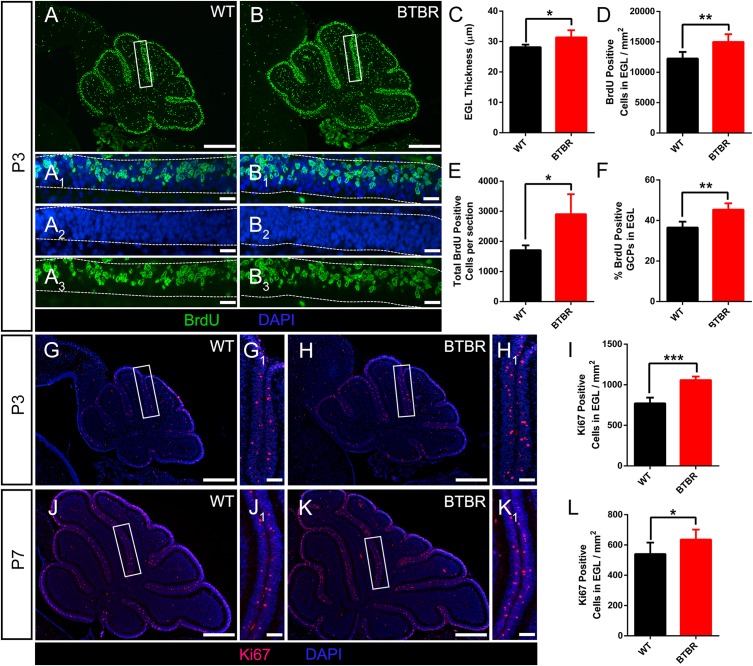
Granule cell precursor proliferation was increased in BTBR mice postnatally. **(A,B)** BrdU staining (green) of sagittal section of cerebellar vermis at P3. Dotted line in **(A_1_–A_3_,B_1_–B_3_)** delimit external granular layer (EGL) where proliferative granule cells originate. Nucleus was counterstained with DAPI (blue). **(C)** Quantification of the EGL thickness showing thicker EGL in BTBR mice at P3 (Student’s *t*-test; *n* = 6,5). **(D)** Quantification of BrdU positive cells per mm^2^ in EGL of each group at P3 (Student’s *t*-test; *n* = 6,5). **(E)** Quantification of total BrdU positive cells in EGL of sagittal section at P3 (Student’s *t*-test; *n* = 6,5). **(F)** Quantification of the percentage of BrdU positive cells in EGL at P3 (Student’s *t*-test; *n* = 6,5). **(G,H)** Ki67 staining (red) of sagittal section of cerebellar vermis at P3 Nucleus was counterstained with DAPI (blue). White panels in **(G,H)** are magnified in **(G_1_,H_1_)**. **(I)** Quantification of Ki67 positive cells per mm^2^ in EGL of sagittal section at P3 (Student’s *t*-test; *n* = 6,5). **(J,K)** Ki67 staining (red) of sagittal section of cerebellar vermis at P7. Nucleus was counterstained with DAPI (blue). White panels in **(J,K)** are magnified in **(J_1_,K_1_)**. **(L)** Quantification of Ki67 positive cells per mm^2^ in EGL of sagittal section at P3 (Student’s *t*-test; *n* = 6,6). All data are displayed as mean ± SD. *P < 0.05, **P < 0.01, ***P < 0.001. Scale bar: **(A,B,G,H,J,K)** 200 μm; **(A_1_–A_3_,B_1_–B_3_)** 10 μm; **(G_1_,H_1_,J_1_,K_1_)** 20 μm.

The foliation pattern divided by fissures of different lengths is a representative morphology of cerebella. The formation is orchestrated by multicellular anchoring centers in which granule cells are the initiating factors and provide the driving physical force (Sudarov and Joyner, 2007). During cerebella development, GCPs in the EGL proliferate and differentiate into granule cells, then gradually mature during migration through the ML to destinations in the IGL. BrdU was used to label the newborn GCPs in the EGL of P3 cerebella (Figures 3A,A_**1**_,B,B_**1**_). Co-staining of nuclei with DAPI revealed that the EGL was much thicker in BTBR cerebella compared to WT (*T*_9_ = 3.218, *P* < 0.05) (Figures 3A_2_,B_2_,C). Simultaneously, the density of BrdU-positive GCPs in the EGL was increased significantly (*T*_9_ = 3.869, *P* < 0.01) (Figures 3A_**3**_,B_**3**_,D). The total GCPs (*T*_9_ = 3.944, *P* < 0.05) (Figure 3E) and proportion (*T*_9_ = 4.899, *P* < 0.01) (Figure 3F) were also increased in BTBR mice. To confirm this result, another marker, Ki67, which is actively expressed during mitosis and degrades soon after caryomitosis, was used. Consistently, the Ki67-positive GCPs in the EGL were multiplied in BTBR mice compared to WT mice (*T*_9_ = 7.827, *P* < 0.001) (Figures 3G–I) at P3. Ki67 was further detected at P7, and it was still much more in BTBR mice than WT mice (*T*_10_ = 2.334, *P* < 0.05) (Figures 3J–L). These results indicate the increased proliferation of GCPs in the cerebella of BTBR mice postnatally was up to P7.

**FIGURE 4:**
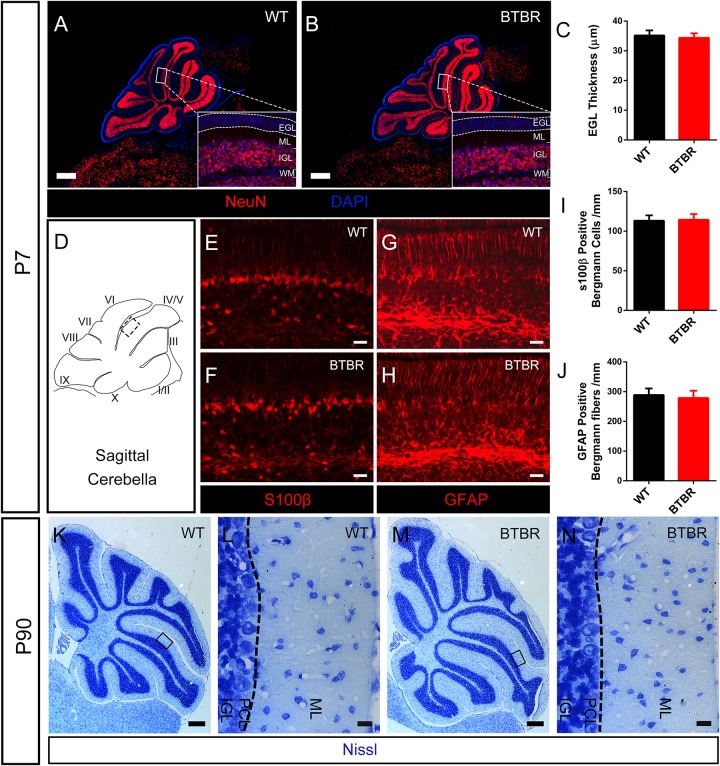
Radial migration of granule neurons in cerebella was not altered in BTBR mice. **(A,B)** NeuN stained (red) granule cells showing mature neurons were all distributed in inner granular layer (IGL) both in WT and BTBR mice at P7 Nucleus was counterstained with DAPI (blue). **(C)** Quantification of EGL thickness in each group at P7. (Student’s *t*-test; *n* = 6,6). **(D)** Schema graph of sagittal cerebella illustrating the observed region (black panel) in figure **(E–H)**. **(E–F)** S100β positive Bergman glia somas in Purkinje cell layer (PCL) at P7. **(G,H)** GFAP positive Bergmann glia fibers in molecular layer (ML) at P7. **(I)** Quantification of Bergman glia somas in PCL per mm (Student’s *t*-test; *n* = 6,6). **(J)** Quantification of Bergman glia fibers in ML per mm. (Student’s *t*-test; *n* = 6,6). **(K–N)** Nissl staining of middle sagittal cerebellar section in adult (P90) WT and BTBR mice. **(L,N)** Are Magnified images of black panels in **(K,M)**. Dotted line in **(L,N)** indicate the boundary between ML and PCL. All data are displayed as mean ± SD. Scale bar: **(A,B,K,M)** 200 μm; **(E–H,L,N)** 10 μm.

Defect in migration of GCs might lead to increased EGL thickness in BTBR mice. We observed that most of the differentiated granule neurons labeled with NeuN staining were located in the IGL in WT mice at P7, with few ectopic neurons in the EGL or ML (Figure 4A). Similarly, no ectopic mature granule neuron was found in BTBR mice (Figure 4B). Notably, the EGL thickness was comparable between the two groups (*T*_10_ = 0.856, *P* = 0.412) (Figure 4C), which indicates that the overproduced granule neurons in BTBR mice migrated efficiently. It was further confirmed that migrating granule neurons identified by slim nuclei in the ML (Yang et al., 2013) were increased in BTBR mice (*T*_10_ = 5.716, *P* < 0.01) (Supplementary Figure S2E), but the proportion or migrating rate was comparable between groups (*T*_10_ = 0.679, *P* = 0.073) (Supplementary Figure S2F). Bergmann glia play a vital role in granule neuron migration. The soma and fibers of Bergmann glia were clearly stained with S100β and GFAP in each group (Figures 4D–H). No aberrations were found in Bergmann glia between groups, neither soma (*T*_10_ = 0.303, *P* = 0.768) (Figure 4I) nor fibers (*T*_10_ = 0.770, *P* = 0.459) (Figure 4J). Furthermore, Nissl staining of 3-month old cerebella revealed conspicuous gross morphological changes in BTBR mice, but the neuron density was similar in the ML between groups (data not shown). The boundary of the IGL was well-defined with no stranded cells (Figures 4K–N), which indicates that the migration of granule neurons was accomplished, in terms of results.

### Purkinje Neurons in BTBR Cerebella Displayed Morphological Hypotrophy With Abnormal Dendritic Spine Formation

**FIGURE 5:**
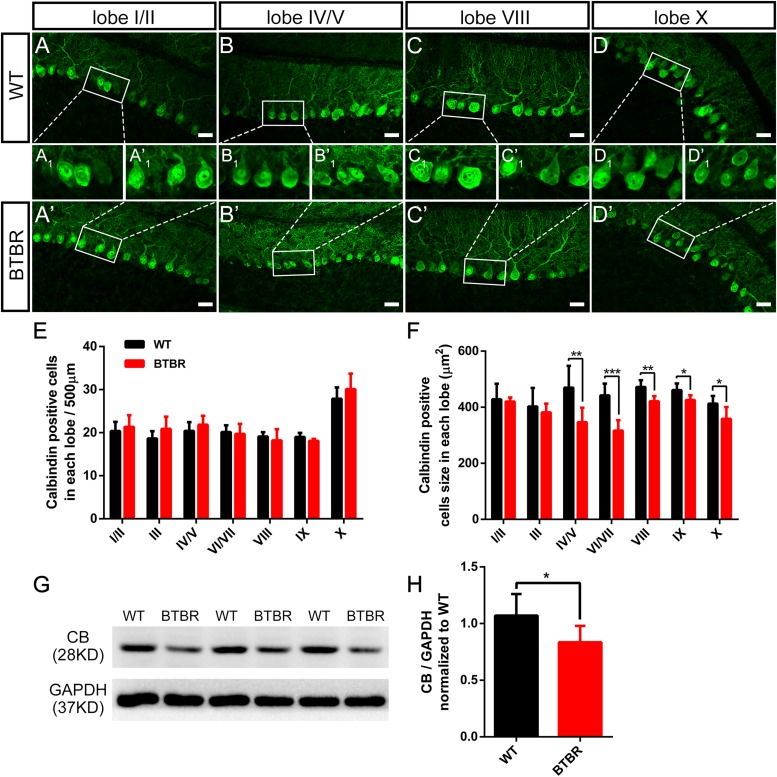
Purkinje neurons’ density did not variate but was significantly hypotrophy in BTBR mice at postnatal day 14. **(A–D)** Calbindin staining Purkinje neurons in cerebellar lobe I/II, IV/V, VIII, X of WT **(A–D)** and BTBR **(A’–D’)** mice at P14. Purkinje neuron somas in white panels are magnified in **(A_1_-D_1_,A’_1_–D’_1_)**. **(E)** Quantification of Purkinje neurons number per 500μm in lobe I-X (Student’s *t*-test; *n* = 6,6). **(F)** Quantification of Purkinje neurons soma size showing hypotrophy in lobe VI-X of BTBR mice. (Student’s *t*-test; *n* = 6,6). **(G)** Representative image of western blotting for calbindin protein in WT and BTBR mice at P14. **(H)** Densitometric quantification of calbindin showing decreased expression in BTBR mice cerebella (Student’s *t*-test; *n* = 6,6). All data are displayed as mean ± SD. *P < 0.05, **P < 0.01, ***P < 0.001. Scale bar: **(A–D)** 25 μm.

Purkinje neurons are the sole efferent neurons in the cerebella and play a key role in motor function. We further investigated whether the Purkinje neurons in the cerebellum of BTBR mice were affected during the critical time when the dystonia-like behavior reached the fastigium at P14. The Purkinje neurons were labeled with the specific marker of calbindin (CB) (Figures 5A–D,A’–D’). At P14, Purkinje neurons were arranged in a monolayer, and the bushy dendrites grew into the ML. The Purkinje neuron density in each lobe was comparable between groups (Figure 5E). However, Purkinje neurons in BTBR cerebella exhibited significant soma hypotrophy compared to WT, especially in the posterolateral lobes, from lobe IV to X (lobe IV/V: *T*_10_ = 3.217, *P* < 0.01; love VI/VII: *T*_10_ = 5.470, *P* < 0.001; lobe VIII: *T*_10_ = 4.142, *P* < 0.01; lobe IX: *T*_10_ = 2.972, *P* < 0.05; lobe X: *T*_10_ = 2.649, *P* < 0.05) (Figures 5A_**1**_–D_**1**_,A_**1**_’–D_**1**_’,F). Western blotting was used to confirm this result (Figure 5G), and calbindin protein expression was decreased in BTBR cerebella (*T*_10_ = 2.416, *P* < 0.05) (Figure 5H), in accordance with the decrease in soma size. We also detected the PCs in adulthood and found a sparse cell distribution with significant cell loss in the posterior lobes in BTBR mice, including lobes VIII (*T*_6_ = 3.019, *P* < 0.05), IX (*T*_6_ = 4.971, *P* < 0.001) and X (*T*_6_ = 2.662, *P* < 0.05) (Supplementary Figure S3). These results indicate that the hypotrophy of Purkinje neurons in postnatal cerebellum of BTBR mice may be a signal of cell death in adulthood.

**FIGURE 6:**
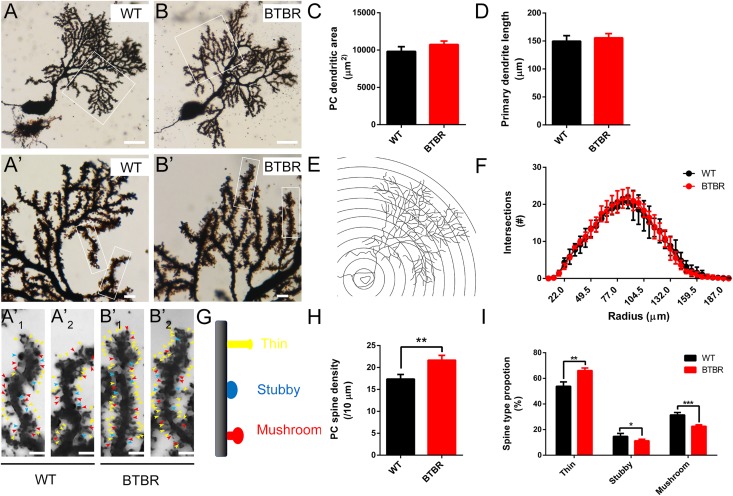
Dendritic spines of Purkinje neurons in BTBR mice were significantly increased with disturbed maturation at postnatal day 14. **(A,B)** Golgi-stained Purkinje neurons in cerebella of WT and BTBR mice. Dendritic branches in white panels are magnified in **(A’,B’)** and furtherly magnified in **(A’_1_,A’_2_,B’_1_,B’_2_)** showing increased and immature dendritic spines in BTBR mice. Yellow, blue, and red arrowheads indicate thin, stubby and mushroom spine types respectively. **(C)** Quantification of Purkinje neuron dendritic profile area in each group. (Student’s *t*-test; *n* = 4,4). **(D)** Quantification of the length of the Purkinje neuron’s primary dendrite in each group (Student’s *t*-test; *n* = 4,4). **(E)** Schema graph showing the method of Sholl analysis. Purkinje neuron’s branches are incised by concentric circles with 5.5 μm radius steps from the soma. **(F)** Quantification of intersections of branches and circles at different radius showing similar level of dendrite arborization in WT and BTBR mice (Two-way repeated measure test; *n* = 4, 4 mice). **(G)** Schema graph illustrating the spine maturity progresses (up to down) from long thin structure (yellow) to transitional stubby (blue) and mushroom mature form (red). **(H)** Quantification of dendritic spines of Purkinje neurons per 10 μm branch showing increased spine density in BTBR mice (Student’s *t*-test; *n* = 4,4). **(I)** Quantification of the percentage of each spine type showing immature development trend in BTBR mice (Student’s *t*-test; *n* = 4,4). All data are displayed as mean ± SD. *P < 0.05, **P < 0.01, ***P < 0.001. Scale bar: **(A,B)** 25 μm; **(A’,B’)** 5μm; **(A’_1_,A’_2_,B’_1_,B’_2_)** 2 μm.

Golgi staining was used to examine the dendrites of PCs at P14 (Figures 6A,B,A’,B’). Representative images showed no differences in gross morphology of Purkinje neurons between groups. There were no abnormalities in extended areas of dendrites (*T*_6_ = 2.315, *P* = 0.060) (Figure 6C), primary dendrite length (*T*_6_ = 0.951, *P* = 0.378) (Figure 6D), or the complexity of the dendrite arborization as assessed by Sholl analysis [*F*(1,204) = 0.105, *P* = 0.757] (Figures 6E,F) of BTBR mice. However, spine density of PCs in BTBR cerebella was significantly increased (*T*_6_ = 5.793, *P* < 0.01) compared to WT controls, with a close array in dendritic branches (Figures 6A’_**1**_-B’_**2**_,H). The Dendritic spines exhibit a transformed morphology during their development and maturation, which reflects different synaptic function at different stages (Nimchinsky et al., 2002). The spines are classified into three subtypes, thin, stubby and mushroom (Figure 6G), and the maturation of the spines was generally assessed. The proportion of the immature long, thin subtype was significantly increased (*T*_6_ = 6.147, *P* < 0.01) in BTBR mice compared to WT mice, and the transitional stubby subtype (*T*_6_ = 2.617, *P* < 0.05) and mature wide-headed mushroom spines (*T*_6_ = 7.738, *P* < 0.001) were decreased (Figures 6A’_**1**_-B’_**2**_,I). These results demonstrate that the dendrite spine formation of PCs was strongly promoted but the mature process was retarded in BTBR mice.

### *TRPC* Genes Were Involved in the Impaired Cerebellar Development in BTBR Mice

**FIGURE 7:**
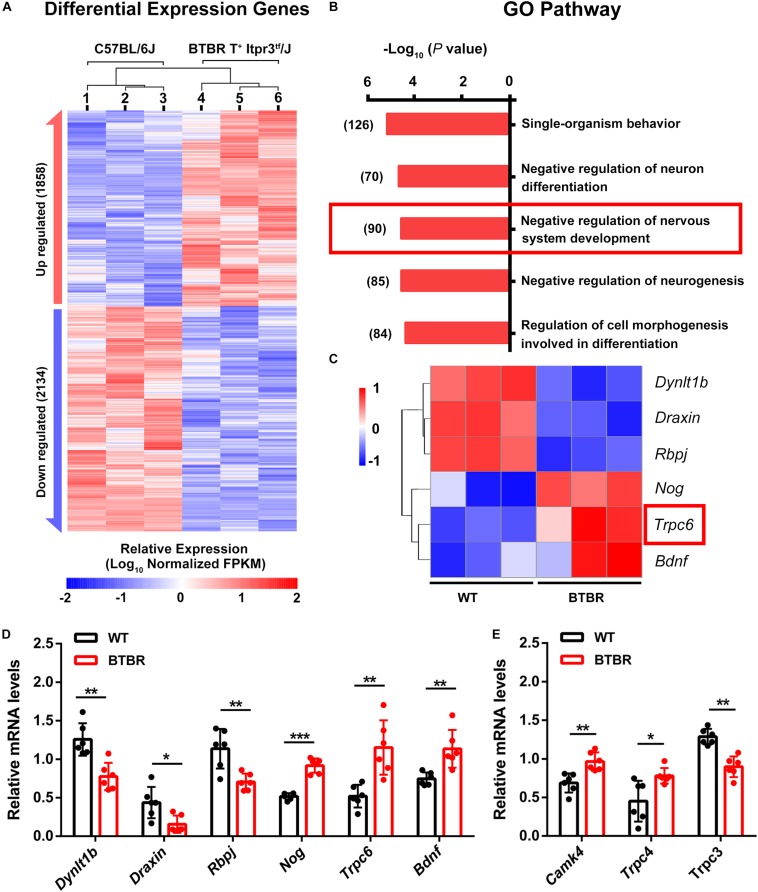
*Trpc* gene differential expression disturbed cerebellar development in BTBR mice at postnatal day 14. **(A)** Hierarchical clustering of differential expression genes in cerebella of C57BL/6J and BTBR mice at postnatal day 14. **(B)** Enriched top five GO pathway in biological process. The former significant pathway involved in cerebella development is highlighted in red frame. Number of differentially expressed genes in each pathway is list side to the bar. **(C)** Heat map depicting 6 significant genes identified from highlighted pathway in B with PPI by STRING (Supplementary Figure S2). *Trpc6* is highlighted in red frame. **(D)** Validation of gene expression in control and BTBR mice by real time PCR. **(E)** Validation of *Trpc3*, *Trcp4* and *Camk4* genes expression in control and BTBR mice by real time PCR.

To examine the underlying mechanism of the abnormal cerebellar development in BTBR mice, we performed RNA- seq in whole cerebella tissue of WT and BTBR mice at P14. We identified 3992 differentially expressed genes (*P* < 0.05) in BTBR mice, with 1858 upregulated and 2134 downregulated (Figure 7A). For functional annotation, the GO term enrichment of differentially expressed genes was analyzed in biological processes. The top-ranking pathways were primarily involved in central nervous system development, neurogenesis, differentiation, cell development and morphogenesis (Figure 7B), which are highly consistent with the abnormal development of the cerebella in BTBR mice. The pathway of negative regulation of nervous system development was noticeable, which are prominently listed and comprehensive in cerebellar development. After further screening using protein-protein interaction (PPI) networks analysis (Supplementary Figure S4), the critical genes were identified, and significantly increased *TRPC6* was a highly suspicious candidate in BTBR mice (Figure 7C). *TRPC6* is especially expressed during cerebellar development (Huang et al., 2007), and it regulates neurogenesis and synaptic formation (Zhou et al., 2008; Xu et al., 2012). PPI networks (Supplementary Figure S4) indicated the direct interaction of *TRPC6* to the changed allele, *Itpr3*, of BTBR mice, suggesting its critical role. The expression was verified using RT-PCR (Figure 7D). We simultaneously detected *CAMK IV* gene expression, which acts downstream of *TRPC6*, and found that it was upregulated as expected (Figure 7E). *TRPC3* and *4* were also detected and exhibited decreased and increased expression, respectively, consistent with the RNA-seq results (Figure 7E). Therefore, the RNA-seq suggests that dysregulated *TRPC* expression might contribute to the impaired cerebellar development and might result in more serious disorders over time.

## Discussion

We demonstrated that BTBR mice exhibited severe infancy-onset dystonia-like behaviors with significant impairments in motor coordination and motor learning, which were also observed in patients with ASD. These motor dysfunctions were highly linked to the abnormal development of the cerebellum. The emerging of dystonia-like behavior in BTBR mice coincided with an increasing proliferation of GCPs, which gave rise to enlargement of the EGL in cerebella and enhanced foliation. Besides, Purkinje neurons in BTBR mice were found to have somatic hypotrophy, with increased dendritic spine formation but suppressed spine maturation. *TRPC* was suggested to be responsible for the impaired neurodevelopment and further motor dysfunction. Our observations demonstrated that disturbed cerebellar neurogenesis occurred during the comorbidities of ASD and movement disorders, and attention should be paid to the key role played by *TRPC* protein family for further study and future approaches of therapy.

Motor dysfunction represents a heterogeneous array of non-diagnostic symptoms in ASD. In this study, we identified dystonia-like behavior, such as hyperflexion, clasping and twisting, eliciting by tail suspension in BTBR mice. The abnormal hindlimb clasping or twisting in BTBR mice also caused a defect to hang from a wire grid. Additionally, impaired fine motor skills in ASD patients are highly linked with social symptomatology (LeBarton and Iverson, 2013). In agreement with these findings, we report here that BTBR mice exhibited skilled walking pattern deficits using the regularly and irregularly spaced horizontal ladder test. The rotarod task is often regarded as a test of cerebellar coordination and motor ability. WT mice showed better performance on the rotarod task across the consecutive learning test, while BTBR mice exhibited slower learning which might due to the inattention.

Postmortem and functional imaging studies widely identified the cerebellum as one of the most important brain regions associated with motor deficits in ASD patients (Wang et al., 2014; Hampson and Blatt, 2015). The onset of motor deficits in BTBR mice coincided with the critical period of cerebellar development, suggesting abnormalities in the cerebellum as the neural substrate of motor impairments. Haijie Yang reported an increase in cerebellar foliation and larger gross brain volume of BTBR mice (Yang et al., 2015). In agreement with this finding, we found that increased cerebellar size and IGL area were obvious compared to the WT mice, with microscopic enhanced foliation. The phenotype was meaningful, for that folia in the cerebellum serve as a broad platform for organizing cerebellar circuits and be critical in sensory-motor tasks (Sudarov and Joyner, 2007). Welker suggested it as the fundamental unit of sensorimotor integration (Welker, 1990), and disturbed foliation was involved in defects of motor coordination (Le Roy-Duflos, 2001; Chen et al., 2005). Inward accumulation of proliferated GCPs is a pivotal driving force in the cerebellar foliation, and the existing mouse model of disturbed foliation demonstrated an aberrant proliferation of GCPs (Corrales et al., 2006; Wefers et al., 2017). The present study detected increased GCP proliferation and IGL expansion in the BTBR mouse cerebellum. Impaired radial migration was also observed in rodents with increased foliation (Hosaka et al., 2012; Ryan et al., 2017), and a dramatic increase in foliation was credited with a prolonged period of migration of GCPs in human cerebella (Sillitoe and Joyner, 2007). However, Bergmann glia guided migration was not altered in BTBR mice from early postnatal days to adulthood. Thus, the extra lobes of cerebella in BTBR mice are likely arose from over-produced GCPs.

Other reports suggest that PCs also participate in cerebellar foliation (Altman, 1997; Sudarov and Joyner, 2007). PCs anchor the outline of the cortex via axonal projections to the underlying WM at positions that define the base of the fissures. Cerebella of BTBR mouse displayed hypotrophic Purkinje neurons at an early developmental period. The abnormal development of granule cells could ultimately regulate the growth of PCs (Salinas et al., 1994; Shimada et al., 1998; Sadakata et al., 2004), and we inferred that the disrupted patterning of Purkinje cells may be secondary to abnormal GC development. We cannot ignore the fact that PCs are the sole efferent neurons in cerebellum which connect to the outer brain and participate in more complicated neural activity. Abnormal PC development determined the dysfunction of cerebella. Parallel fibers extended by granule neurons in the EGL traveled up and stretched to both sides, being parallel to the pial surface in the ML to connect the Purkinje dendrite. Considering the multiplying granule neurons and invariable even decreased PCs, superabundant incoming of information to an individual PC was predictable. Moreover, the synaptic structure identified by dendritic spines in Purkinje neurons was significantly affected because much more spines existed in a lower matured proportion. Synapses were likely overproduced, but the maturation was suppressed. Immature spines commonly aid in the initiation of synaptic contact (Dunaevsky et al., 1999), and mature spines containing an abundance of neurotransmitter receptors are truly to support synaptic activity (Matsuzaki et al., 2001; Nimchinsky et al., 2002). Abnormal spine formation and maturation would impact the neural circuit and disturb the allomeric function of the brain. Additionally, increasing afferent signal to PCs would inevitably break the physiological balance in transduction, which indicates the critical role played by disturbed information transfer in cerebellar dysfunction.

*TRPC* is a non-selective cation channel that dominantly modulates the Ca^2+^ entry pathway and the release of intracellular Ca^2+^ (Dietrich and Gudermann, 2007). *TRPC3*, *4* and *6* belong to the *TRPC* protein family are particularly expressed in cerebella during the first 6 weeks after birth, at the critical neurogenesis period of the cerebellum, to regulate cerebellar development (Huang et al., 2007).*TRPC3* expression reflects development of the Purkinje cells and *TRPC4* expression is restricted to granule neurons and their precursor. *TRPC6* plays an essential role in G2/M phase transition (Shi et al., 2009), and inhibition or activation of *TRPC6* expression suppresses or accelerates cell growth (Cai et al., 2009; Shi et al., 2009), respectively. Moreover, *TRPC6* participates in the development of dendritic spines and regulates the formation of excitatory synapses in the hippocampus (Zhou et al., 2008), and inhibition of *TRPC6* reduces dendritic arborization and spine density (Tai et al., 2008). The results of RNA-seq analysis indicated that *TRPC* family might be an important regulator involved in the abnormality of cerebellar development of BTBR mice. Moreover, other studies suggest the relationship between *TRPC* and ASD. Wei Li found that *TRPC* signaling was impaired in hippocampal neurons of Mecp2 mutant mice, another ASD mouse model, which led to activity-dependent *BDNF* release disturbances that further accounted for sensory and motor abnormalities (Li et al., 2012). Later, Griesi-Oliveira K demonstrated a reduction or haploinsufficiency of the *TRPC6* gene in ASD individuals, which led to impaired neuronal development, morphology, and function (Griesi-Oliveira et al., 2015). These findings imply *TRPC* genes could be novel predisposing genes for ASD to elucidate the underlying pathophysiology mechanism.

## Conclusion

We demonstrated that abnormal neurogenesis of cerebella in BTBR mice primarily affected foliation and disturbed synaptic formation, which possibly lead to dystonia-like behavior and motor dysfunction. The *TRPC* family was highly indicated as responsible for the impaired cerebellar development and as a novel predisposing gene for ASD. Therefore, *TRPC* should receive more attention and be further explored to elucidate the pathological process of ASD and possible novel treatments.

## Data Availability Statement

The raw data supporting the conclusions of this article will be made available by the authors, without undue reservation, to any qualified researcher.

## Ethics Statement

All of the animal breed and experiments are performed accordance with the National Institutes of Health guide for the care and use of Laboratory animals (NIH Publications No. 8023, revised 1987) and approved by Animal Experiment Committee of Laboratory Animal Center of Army Medical University.

## Author Contributions

RX carried out the experiments, collected and analyzed the data, and wrote the manuscript. HZ, RZ, LW, and ZZ maintained the mice and collected the samples. XL and YM contributed to the quantification and data analysis. XF provided resources and funding, performed the experiments planning, supervised the project, and revised the manuscript.

## Conflict of Interest

The authors declare that the research was conducted in the absence of any commercial or financial relationships that could be construed as a potential conflict of interest.

## Footnotes

1 https://string-db.org/

## Abbreviations

ADHD: attention-deficit/hyperactivity disorder
ASD: autism spectrum disorders
BrdU: 5-bromo-2’-deoxyuridine
CB: calbindin
DAPI: 4’,6-diamidino-2-phenylindole
DCN: deep cerebellar nuclei
DEG: differential expression gene
EGL: external granule layer
FPKM: fragments per kilobase of transcript sequence per millions base pairs sequenced
GCL: granule cell layer
GCP: granule cell precursor
GFAP: glial fibrillary acidic protein
GO: Gene Ontology
HE: Hematoxylin-eosin
IGL: inner granule layer
*Itpr3*: inositol triphosphate receptor 3
ML: molecular layer
NeuN: neuronal nuclei
P10: Postnatal day 10
PBS: phosphate buffered saline
PC: Purkinje cell
PCL: Purkinje cell layer
PFA: paraformaldehyde
PPI: protein–protein interaction
RT: room temperature
SD: standard deviation
TBS: Tris buffered saline
*TRPC*: transient receptor potential canonical channel
WM: white matter
WT: wild type.
