# Supplementary material for: Abnormal Cerebellar Development Is Involved in Dystonia-Like Behaviors and Motor Dysfunction of Autistic BTBR Mice
Source: Front Cell Dev Biol. 2020 Apr 7;8:231. doi: 10.3389/fcell.2020.00231 (PMC7154340; doi:10.3389/fcell.2020.00231)
Supplement: Supplementary file 2 [file Image_1.pdf]

Supplementary figure and figure legend

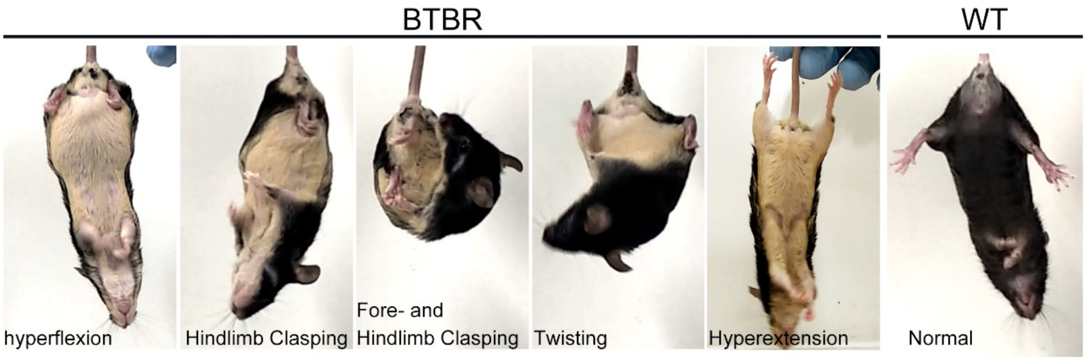

Figure.s1 Adult BTBR mice exhibited typical dystonia-like behaviors.

Representative images of hyperflexion, clasping, trunk twisting and hyperextension as dystonia-like behaviors of BTBR mice in tail suspension test.

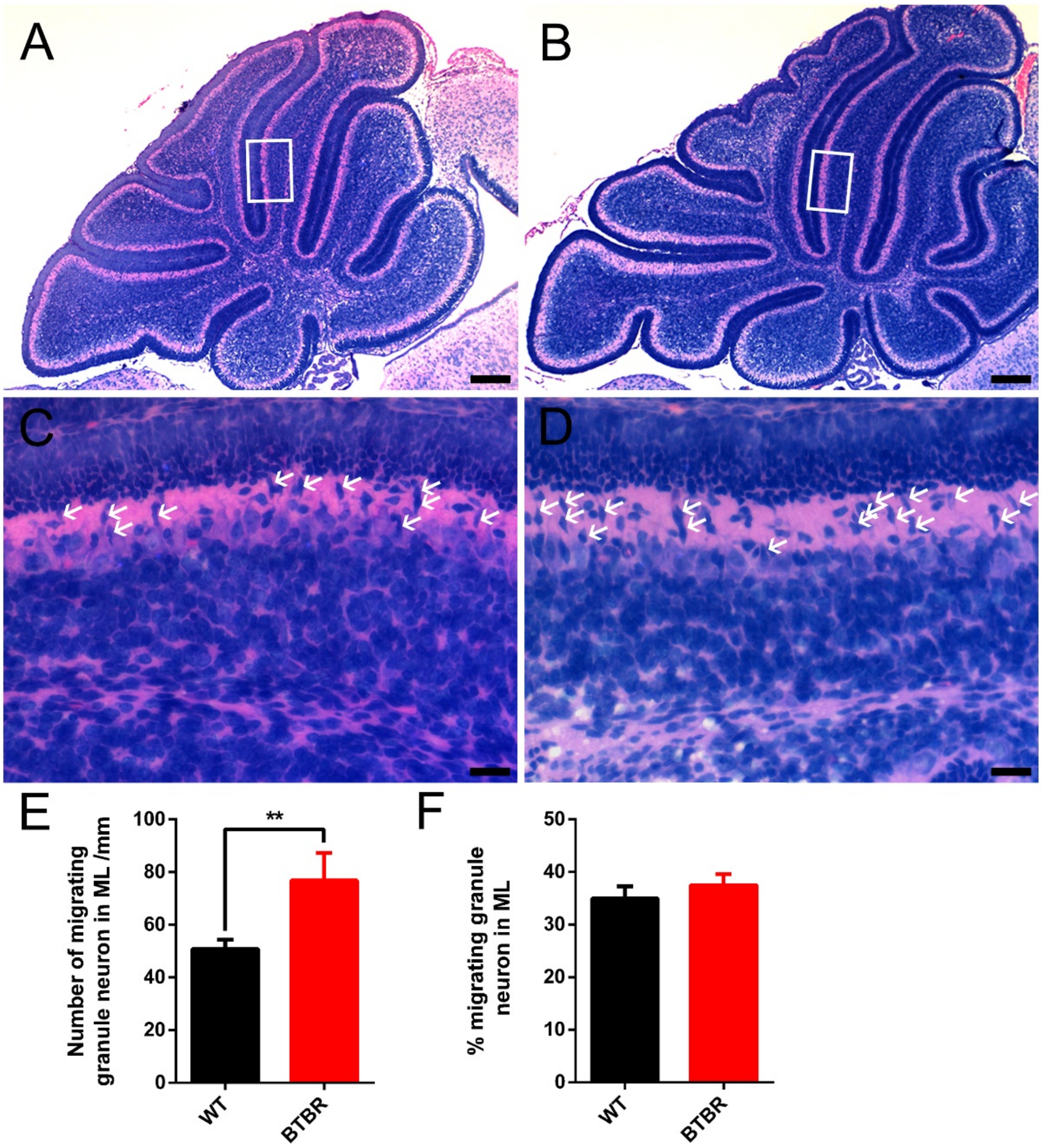

Fig.s2 Migration rate of granule neurons in ML was not affected in BTBR mice at P7.

A-B Hematoxylin-eosin (HE) staining of middle sagittal cerebellar section at postnatal day 7.

C-D Magnification of white box in C, D showing migrating process of granule neurons from EGL to IGL. White arrows indicate the migrating granule neurons identified by the slim nucleus in ML.

E Quantification of number of migrating granule neurons with slim nucleus per mm in

ML. (Student's t-test; n=6,6).

E Quantification of migrating rate of granule neurons in ML. (Student's t-test; n=6,6).

All data are displayed as mean ± SD. **P < 0.01. Scale bar: A, B 200μm; C, D 25μm.

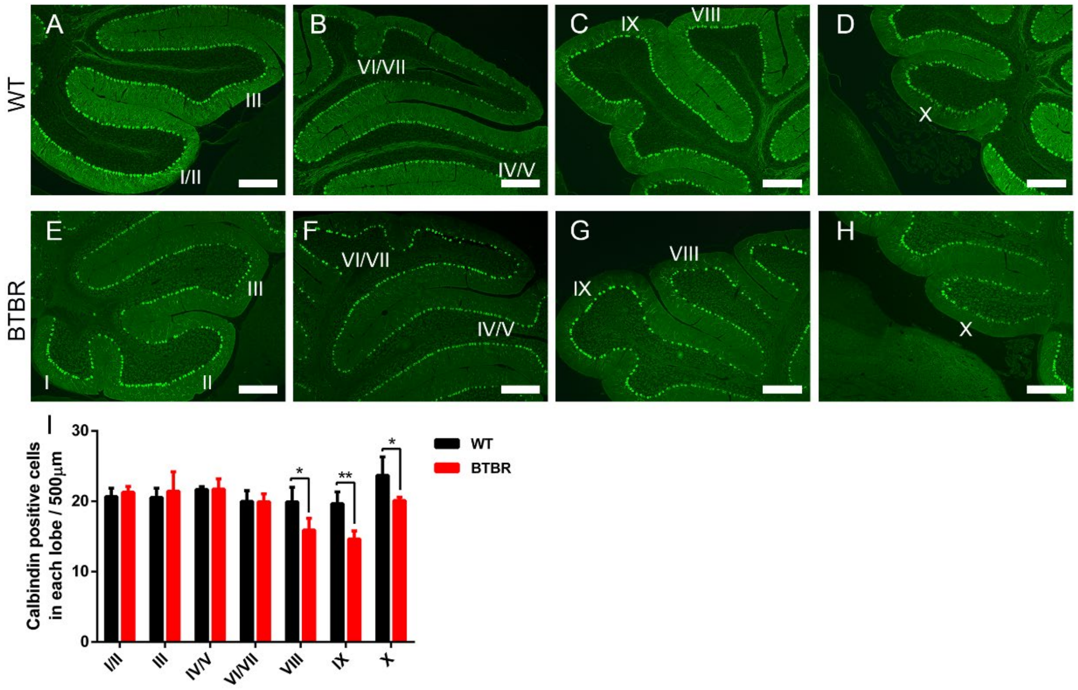

Fig.s3 Purkinje cells significantly lost in BTBR cerebella at adulthood (P90).

A-H Calbindin staining Purkinje neurons in cerebellar lobe I-X of WT (A-D) and BTBR (E-H) mice in adulthood.

I Quantification of Purkinje neurons number per 500μm in lobe I-X (Student's t-test; n=4,4).

All data are displayed as mean ± SD. *P < 0.05, **P < 0.01. Scale bar: A-H 200μm.

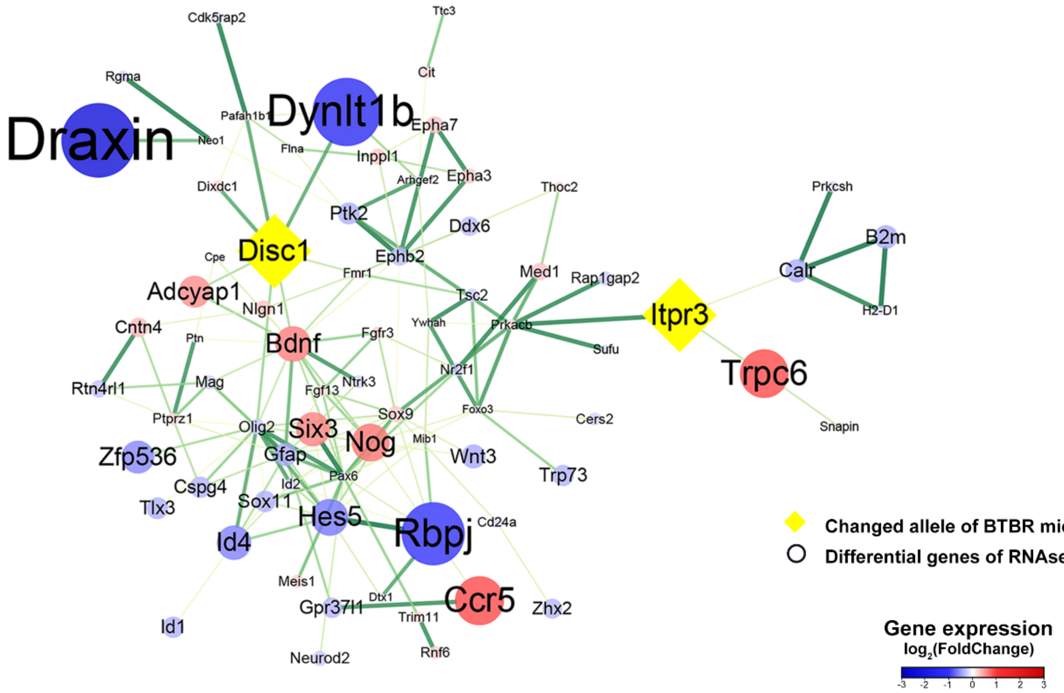

Fig.s4 Protein-protein interaction (PPI) networks identified by STRING of selected 90 proteins in Negative regulation of nervous system development pathway.

Differential expression genes classified in upregulated (red) and downregulated (blue).

Changed alleles of BTBR mice are added and highlighted. Disconnected nodes were hide in the network.
