# Supplementary material for: Abnormal Cerebellar Development Is Involved in Dystonia-Like Behaviors and Motor Dysfunction of Autistic BTBR Mice
Source: Front Cell Dev Biol. 2020 Apr 7;8:231. doi: 10.3389/fcell.2020.00231 (PMC7154340; doi:10.3389/fcell.2020.00231)
Supplement: Supplementary file 3 [file Table_1.pdf]

Supplemental Table: The sequence of primers used in this study

| Gene | Primer sense (5'-3') | Primer antisense (5'-3') |
|---|---|---|
| *Dynlt1b* | AAAGCGCCATCGGTGGTAAT | GGTCTTGTTCTCCCATCGGAC |
| *Draxin* | GTCCCAGGCAAGCCATCAT | CGGGGTAGGGCAAAGTCAG |
| *Rbpj* | AACAGCGATGACATTGGTGTG | ACCGAAGGCGATTGAACAGTG |
| *Nog* | GGTGTGTAAGCCATCCAAGTCT | CCGAGTTCTAGCAGGAACACTTAC |
| *Trpc6* | GGTGCGGAAGATGCTAGAAG | AGCTGGATGGTTGAGGATTG |
| *Bdnf* | GGGTCACAGCGGCAGATAAA | GCCTTTGGATACCGGGACTT |
| *Camk4* | GAGAACCTCGTCCCGGATTAC | ACACAATGGATGTAGCACCCC |
| *Trpc3* | AAAGAAAACGATGAGGTGAATGAAG | CATAACGAAGGCTGGAGATATCCT |
| *Trpc4* | GTGTGCTACCTGATAGCTCCC | GGCAGAGACACGTTCGTTATT |
| *Gapdh* | TGGGTGTGAACCACGAGAAA | AAAGTTGTCATGGATGACCTTGG |
